# Evolutionary history of *Mycobacterium leprae* in the Pacific Islands

**DOI:** 10.1098/rstb.2019.0582

**Published:** 2020-10-05

**Authors:** Kelly E. Blevins, Adele E. Crane, Christopher Lum, Kanako Furuta, Keolu Fox, Anne C. Stone

**Affiliations:** 1 School of Human Evolution and Social Change, Arizona State University, Tempe, AZ, USA; 2 Center for Bioarchaeological Research, Arizona State University, Tempe, AZ, USA; 3 School of Life Sciences, Arizona State University, Tempe, AZ, USA; 4 Center for Evolution and Medicine, Arizona State University, Tempe, AZ, USA; 5 Department of Pathology, John A Burns School of Medicine, University of Hawaii, Honolulu, HI, USA; 6 Hawaii Pathologists Laboratory, Honolulu, HI 96813, USA; 7 Departments of Anthropology and Global Health, University of California, San Diego, CA, USA

**Keywords:** *Mycobacterium leprae*, whole genome, leprosy, Hansen's disease, Pacific Islands, FFPE

## Abstract

As one of the oldest known human diseases, leprosy or Hansen's disease remains a public health concern around the world with over 200 000 new cases in 2018. Most human leprosy cases are caused by *Mycobacterium leprae*, but a small number of cases are now known to be caused by *Mycobacterium lepromatosis*, a sister taxon of *M. leprae*. The global pattern of genomic variation in *M. leprae* is not well defined. Particularly, in the Pacific Islands, the origins of leprosy are disputed. Historically, it has been argued that leprosy arrived on the islands during nineteenth century colonialism, but some oral traditions and palaeopathological evidence suggest an older introduction. To address this, as well as investigate patterns of pathogen exchange across the Pacific Islands, we extracted DNA from 39 formalin-fixed paraffin-embedded biopsy blocks dating to 1992–2016. Using whole-genome enrichment and next-generation sequencing, we produced nine *M. leprae* genomes dating to 1998–2015 and ranging from 4-63× depth of coverage. Phylogenetic analyses indicate that these strains belong to basal lineages within the *M. leprae* phylogeny, specifically falling in branches 0 and 5. The phylogeographical patterning and evolutionary dating analysis of these strains support a pre-modern introduction of *M. leprae* into the Pacific Islands.

This article is part of the theme issue ‘Insights into health and disease from ancient biomolecules’.

## 1. Introduction

Leprosy, or Hansen's disease, is a chronic and progressive infectious disease caused by the obligate intracellular pathogen *Mycobacterium leprae* and the more recently discovered *Mycobacterium lepromatosis* [1,2]*.* The *M. leprae* genome is compact and demonstrates a low level of diversity, with less than 300 single nucleotide polymorphisms (SNPs) segregating geographically disparate strains [3–5]. In initial comparative genetic studies, Monot *et al*. [6] compared the reference TN (India) genome sequence to 142 kb of sequence from a Brazilian strain (Br4923) and found five SNPs; of these, three SNPs were used to identify and define *M. leprae* types (SNP types 1–4) in a broader range of strains. Subsequent comparative analyses resulted in the identification of additional SNP subtypes (A-P) [7] that have been used for broader genotyping studies (e.g. [8–10]).

More recently, phylogenies generated from genome data were used to define six branches (denoted by 0–5) [3,4,11]. As with the SNP subtypes, the branches have geographical associations, with branch 0 found mainly in Eastern Asia, branch 1 found mainly in Southern and Eastern Asia, the paraphyletic branch 2 found in the Near East and South Asia, branch 3 primarily found in North and Latin America, branch 4 found mainly in West Africa and South America, and branch 5 found in the Pacific Islands [3,7]. Monot *et al*. [7] suggest that the geographical patterning of strains reflects human migration patterns and trade along early and more modern routes, including the Silk Road. Despite close geographical associations of branches, new genomic data and recent work adding to the understanding of ancient genetic diversity [3,11], the origin and dispersal of *M. leprae* is still not well understood.

Leprosy was endemic in many Western Pacific and Pacific Island countries prior to 2010 [12,13]. Today, the success of public health campaigns and effectiveness of multidrug therapy are evident; now most of the Pacific Islands have prevalence rates below the elimination threshold [14,15]. The antiquity of the disease in the region, however, is not well documented by historical or palaeopathological sources. While cases caused by *M. lepromatosis* have not been identified in the Pacific Islands, two previously published whole *M. leprae* genomes from the region belong to the deepest lineages 0 and 5 [4], and genotyping studies have identified the presence of all *M. leprae* SNP types in New Caledonia [6,9]. Unfortunately, genomic data are limited for strains present in the region, precluding a clear understanding of the diversity and evolutionary history of this pathogen.

## 2. Antiquity of *Mycobacterium leprae* globally and in the Pacific Islands

The oldest probable skeletal evidence of leprosy comes from Balathal, India, dating to 2000 BCE [16], although there is a skeleton from Europe with possible rhinomaxillary signs of leprosy dating to 1500 years earlier [17]. The oldest written record of leprosy is described in the *Suchruta samhita*, an Indian text from around 600 BCE (described in [18,19]). Later evidence of leprosy in Southeast Asia comes from a skeleton in Thailand, dating to 200 BCE–300 CE [20]. Skeletal and biomolecular evidence of leprosy from around the first to third century CE has also been identified in Central Asia [21,22]. Lack of molecular evidence from these early skeletal cases, however, makes confirmation of the disease difficult.

More recent discussion of *M. leprae* population history has focused on entry into regions, specifically Western Eurasia, rather than global patterning. Donoghue *et al*. [23] argue that westward migration of groups from Central Asia in the first millennium may have introduced different *M. leprae* strains to Europe, whereas Schuenemann *et al*. [3] offer two models for the high level of genetic diversity in medieval Europe. Either *M. leprae* originated in Western Eurasia or diverse strains of *M. leprae* with different geographical origins were introduced to Europe prior to the Medieval period [3]. Recent efforts to fill in the history of *M. leprae* have introduced many more ancient lineages to the family tree, however these ancient isolates are largely limited to Western Europe and Eurasia [3,11].

Despite the considerable number of palaeopathological investigations in the Pacific Islands, particularly on the United States territory of Guam (see [24]), there is little published evidence of premodern skeletal leprosy. This could be owing to its mis-diagnosis as treponemal disease [25], compounded by the poor preservation of hand and foot bones—important for identifying skeletal leprosy—in tropical environments. The oldest convincing skeletal evidence of leprosy from the Pacific Islands comes from the Mariana Islands. Trembly [26] describes six skeletons with destruction of the metatarsals, metacarpals, and manual and pedal phalanges, including concentric atrophy of the diaphyses, indicating probable leprosy infection [27]. One of these individuals dates to 830 ± 170 CE, and the three others for which radiocarbon dating was performed, date between 1140 and 1370 CE, suggesting the disease was present in the Pacific Islands prior to Portuguese and Spanish contact in the 1500s.

The earliest skeletal evidence of leprosy in Japan is later, with the oldest known skeletal case dated to the medieval period (1200–1400 CE) [28]. Suzuki *et al*. [29,30] present skeletal and biomolecular evidence of leprosy infection from later time periods (1400–1800 CE). Historical evidence of leprosy in Japan, however, can be traced back to the eighth century CE in the *Chronicles of Japan* (*Nihon Shoki*) (described in [31]). Called *rai*, the disease description matches that of leprosy and was said to have been introduced by a migrant from the Korean peninsula [31, p. 20].

Following the World Health Organization's regional country designations [15], the earliest historical and skeletal evidence of leprosy from the Southeast Asia region and the continental portion of the Western Pacific region predates the earliest evidence from the Western Pacific Islands. While there is no published skeletal evidence of leprosy from premodern China, a passage from the medical text *Lingshu* describes signs consistent with leprosy, such as collapse of the nose, leg deformity, eyebrow loss, and hoarseness [32]; this text has been used to establish the presence of leprosy in China before the first century CE [33, pp. 19–25].

Based on the lack of diagnostic evidence and unclear descriptions of signs and symptoms, some historians and physicians have concluded that leprosy was introduced to the Pacific Islands during the nineteenth century, first in Hawaii and then to other Pacific Islands by Chinese migrants [34,35]. Native Hawaiians report that a mysterious disease arrived in the Hawaiian Islands from China, brought by Chinese sailors or by native Hawaiians who had been sent to China on trade missions. They call the disease *ma‘i pake*, meaning ‘Chinese disease' and *m‘ai ali‘i*, meaning ‘chief's or royal disease' [36,37]. It has been argued that an uptick in leprosy cases occurred across the Pacific Islands during the 1800s [38]; this is probably a bias introduced by Western physicians diagnosing the disease and geopolitical pressure to isolate those affected on island colonies, for example Kalaupapa on Molokai or Makogai in Fiji [39]. Because the convincing skeletal evidence of leprosy on the Mariana Islands by 800 CE [26,40] is limited, it remains unresolved whether *M. leprae* was introduced during the initial island migrations or later during subsequent periods of colonialism and imperialism [41].

To further the understanding of the origins of *M. leprae* in the Pacific Islands as well as the phylogeography and genetic diversity of strains in an undersampled region, we present phylogenetic and evolutionary dating analyses of nine *M. leprae* genomes from Samoa (*n* = 2), Hawaii (*n* = 5), and Guam (*n* = 2) isolated from formalin-fixed paraffin-embedded (FFPE) tissue samples. Such archival tissue specimens are valuable resources for clinical genomics, however, fragmentation and low DNA yield can make sufficient recovery difficult [42]. We applied techniques used more commonly on ancient DNA, and after analyses of the genomic data, we determined that these isolate lineages fall into branches 0 and 5, which form the deepest lineages of the *M. leprae* family tree. These data also support a strong geographical association of modern lineages, with a temporal and geographical distribution that indicates an introduction of the pathogen during the peopling of the Pacific Islands.

## 3. Material and methods

### (a) Sampling

Data and samples were retrospectively collected from the pathology database at Hawaii Pathologists Laboratory, Honolulu, HI. Biopsies were pathologically diagnosed with leprosy between 1991 and 2016. In order to focus on the Pacific Islander population, presumptive Hawaiian or Polynesian ethnicities were determined by the patient's first or last name. Thirty-nine FFPE samples from Pacific Islanders were obtained for this study. Patient samples were received throughout the Austronesian region; Oahu (*n* = 18), Hawaii neighbour islands (*n* = 6), Guam (*n* = 3), Saipan (*n* = 2), American Samoa (*n* = 6), Marshall Islands (*n* = 2) and Palau (*n* = 2). There were 21 males and 18 females. The study population ranged from 9 to 79 years of age with an average age of 34 years old.

### (b) DNA extraction and quantitative real-time polymerase chain reaction analyses

Thirty-nine FFPE tissue biopsies from 34 people were sectioned using a microtome; one to three 40–46 µm slices were removed from the FFPE block. In some cases, almost all of the sample was used. Other genetic studies using FFPE blocks removed 2–10 slices at 5–20 µm per slice. We chose to use fewer but thicker slices so that we could manually excise the tissue sample from the paraffin, thereby reducing the amount of paraffin in the extraction reaction and inhibition in downstream polymerase chain reaction (PCR). Excess paraffin was manually removed from each slice using a scalpel blade, and extractions were performed directly on the excised tissue samples. The microtome blade and scalpel were sterilized with a 10% bleach solution, followed by water and 70% ethanol between sampling of each FFPE block.

DNA extractions were performed on 28 FFPE block samples following Purification of Total DNA from Animal Tissues protocol from the Qiagen DNeasy Blood and Tissue (DBT) kit with no FFPE-specific pre-treatments (following [43]) and some modifications: lysis occurred for 24 h with intermittent vortexing, Qiagen MinElute spin columns were used in place of the DBT spin columns, and DNA was eluted twice in 100 ul of Tris EDTA buffer with 0.05% Tween-20 (TET). Owing to low yields, further extractions were performed using a protocol developed for extracting ancient DNA with a modified digest step (DAB) [44,45]. DAB was used to extract DNA from 19 samples, including 11 new samples and eight of the samples previously extracted using DBT. To improve yields further, the DAB protocol was modified to include a 15 min heat treatment at 98°C during digestion and prior to the addition of proteinase K, following Gilbert *et al*. [43].

All extracts, 1 : 10 dilutions of the extracts, and extraction blanks were screened for *M. leprae* DNA in triplicate using TaqMan quantitative real-time PCR (qPCR) assays designed to target two *M. leprae*-specific elements: *85B* and RLEP. The qPCR assays amplify an 80 bp fragment of the single-copy gene *85B* [46] and a 70 bp region of the multicopy repeat element RLEP [47]. Samples were chosen for whole-genome capture based on how many replicates amplified for both assays.

### (c) Library preparation and whole-genome capture

Fragment shearing was not necessary owing to the already short fragment lengths (less than 300 bp). The 8 DBT and 11 DAB extracts that had the best qPCR signals were converted into double-indexed libraries following Meyer & Kircher [48]. Nucleotide misincorporation patterns were identified in the DBT sequencing data, so the 11 DAB extracts were treated with uracil-DNA glycosylase (UDG) during library preparation. For the DBT libraries, 35–290 ng were enriched for the *M. leprae* genome using an Arbor Biosciences myBaits kit V 3.02. The in-solution capture reaction used biotinylated baits prepared from *M. leprae* Br4923, Thai53, and NHDP strains. After a 48 h hybridization reaction, capture products were amplified for 14 cycles using AccuPrime™ Pfx DNA polymerase. The DBT enriched libraries, extraction blank, and library blank were sequenced on the Illumina HiSeq2500 with 2 × 100 cycles. For the DAB libraries, 542–2890 ng were enriched for the *M. leprae* genome using the myBaits kit V 3.02 with the same bait preparation as above. The enriched DAB libraries, extraction blank and library blank were sequenced with 2 × 75 cycles across two Illumina Mi-Seq runs.

### (d) Data acquisition and processing

Owing to their low depth of coverage after DBT extraction, samples 511, 515, 519 and 523A1 were re-extracted using DAB, re-captured and re-sequenced. The quality of the raw data for these four samples from both sequencing runs was visualized using fastqc v.0.11.7 [49] and determined to be of high enough and consistent quality to concatenate, thus increasing genome coverage and depth of coverage. Paired-end reads from all samples were trimmed and merged using AdapterRemoval v.2 v.2.2.3, with the following parameters – minquality 20 – minlength 30 [50]. The resulting merged reads were mapped to the *M. leprae* TN strain (NCBI NC_002677.1) using Burrows-Wheeler Alignment tool (BWA) v.0.7.17 with the following commands and parameters: aln samse -l 1000 -n 0.1 [51]. Unmapped reads were removed using samtools view and PCR duplicates were removed using samtools markdup -r v.1.9 [52]. Given that samples not treated with UDG exhibited terminal misincorporations (electronic supplementary material, figures S1 and S2), MapDamage 2.0 (v.2.0.9) was used to rescale the quality scores for terminal bases prior to variant calling [53]. Coverage and mapping quality estimates were generated using QualiMap 2 (v.2.2.1) [54].

For comparative purposes, genomic data for 164 modern and ancient *M. leprae* samples were downloaded from the NCBI Sequence Read Archive in FASTQ and FASTA format (electronic supplementary material, table S1). Raw fastq data were downloaded using SRA Toolkit v. 2.8.0 (https://github.com/ncbi/sra-tools) fastq-dump command. The 164 comparative samples used in this analysis do not include published samples with an average depth of coverage of less than 5×. Samples that have been identified as hypermutators were included in the maximum-likelihood and maximum-parsimony phylogenetic trees but not in the BEAST analysis [4]. Ancient sample data were processed exactly as the study samples. Modern sample data were processed similarly to the above samples, except that paired-end reads were not merged, and the mapping qualities were not rescaled. Assembled genomes in FASTA format, TN, Br4923, Kyoto-2 and *M. lepromatosis* JRPY were mapped to the reference genome using bwa mem with default parameters [55].

### (e) Variant calling and analysis

SNPs were called for all samples using GATK UnifiedGenotyper v. 3.5 with default parameters except for – out_mode EMIT_ALL_SITES [56]. The resulting VCF files were filtered for homozygous SNPs with a coverage depth of at least 5× and a GATK genotyping quality of at least 30 and aligned using MultiVCFAnalyzer v.0.85.1 (https://github.com/alexherbig/MultiVCFAnalyzer/releases) [57]. Also using MultiVCFAnalyzer, positions that are in known repeat regions and that were covered in the SK12 negative control were excluded, following Honap *et al*. [58] and Schuenemann *et al*. [11]. SNPs in the *M. lepromatosis* outgroup were only called if they were also present in *M. leprae*. For the full genomes, the VCF files were manually edited for a coverage depth of greater than 5× and a genotyping quality of at least 30 so that they could be filtered simultaneously with the raw fastq data. An alignment of 3521 SNPs was generated; after removing sites with less than 95% coverage across the dataset, a final alignment of 2736 SNPs was used for phylogenetic analyses. After removing genomes previously identified as hypermutators [3] and sites with less than 95% site coverage, a final alignment of 2184 SNPs was used for BEAST [59] analysis.

To investigate the effects of the unique SNPs in our samples that are shared among the Pacific Island strains, the VCF files for the samples generated in this study were processed using SnpEff v.3.1 to annotate variants and determine their functional effects [60]. Default parameters were used, except the upstream and downstream interval size was set to 100. SnpEff v.3.1 was used to ensure compatibility with the MultiVCFAnalyzer SNP table output.

### (f) Phylogenetic analysis

To examine consistency in topology across different methods, two phylogenetic trees were made. A maximum-likelihood tree was constructed using the GTR nucleotide substitution model with the GAMMA model of rate heterogeneity (-m GTRGAMMA) with 1000 bootstrap replicates using RAxML v.8.2.12 [61]. A maximum-parsimony (MP) tree was made using the subtree-pruning-regrafting inference model and a bootstrap test of phylogeny with 1000 replicates in MEGA7 [62]. All trees were rooted using *M. lepromatosis* as the outgroup.

We also estimated the time to most recent common ancestor (tMRCA) and substitution rates through application of Bayesian methods using BEAST 2.4.5 [59]. A concatenated SNP alignment of 2184 informative sites was used in the analysis. Additionally, counts of invariant sites shared by all strains and the reference were included to avoid ascertainment bias of only using variant sites. The alignment was run through the jModelTest2 tool [63,64] to determine a best-fit model of nucleotide substitution. The alignment was analyzed using a Bayesian Skyline model, assuming a variable population size [59] with a relaxed clock and a GTR substitution model [65,66]. The alignment was also run again using a strict clock with a GTR substitution model and a lognormal relaxed clock with an HKY substitution model. Each run was completed with 300 000 000 iterations and a 30 000 000 burn-in. Calibrated radiocarbon dates were used for ancient strain tip dates and sampling/isolation dates were used for modern samples.

## 4. Results

### (a) DNA extraction and screening

DNA extraction for DBT and DAB [44,45] methods yielded between less than 0.01 ng ul^−1^ and 18.2 ng ul^−1^. For the initial 28 DBT extractions, the yields were between less than 0.01 ng ul^−1^ and 0.7 ng ul^−1^. Owing to these low yields, further extractions were performed on 19 samples, eight of which had also been extracted using DBT. The DAB extraction yields were between 0.516 and 18.2 ng ul^−1^. All samples and 1 : 10 dilutions of each sample underwent qPCR assays in triplicate for *85B* and RLEP regions. Samples with all three replicates amplifying for at least one assay were considered for whole-genome capture, including assays of 1 : 10 diluted extracts (table 1). In addition to the number of replicates amplified, the mean number of cycles of amplification, DNA quantity, and leprosy type were evaluated before a subset of samples was selected for whole-genome capture and sequencing (electronic supplementary material, table S2).

**Table 1.** Summary of DNA extraction concentrations and qPCR assay results for samples sequenced in this study. (The number of successfully amplified replicates out of three is given for each assay.)

sample ID	type	DNA extraction type	DNA concentration (ng ul^−1^)	*85B*	*85B* 1 : 10	RLEP	RLEP 1 : 10
511	LL	DBT	0.414	3/3	2/3	3/3	3/3
		DAB	6.68	0/3	0/3	3/3	3/3
515	LL	DBT	0.198	3/3	1/3	3/3	3/3
		DAB	9.96	0/3	0/3	1/3	3/3
516	LL	DBT	<0.01	3/3	3/3	3/3	3/3
		DAB	2.56	0/3	0/3	3/3	3/3
517	LL	DBT	0.436	3/3	3/3	3/3	3/3
		DAB	6.9	0/3	0/3	3/3	3/3
518	BL	DBT	0.67	3/3	3/3	3/3	3/3
		DAB	1.56	0/3	0/3	0/3	3/3
519	BL	DBT	0.26	3/3	3/3	3/3	3/3
		DAB	6.06	0/3	0/3	0/3	3/3
520	BL	DBT	0.138	3/3	3/3	3/3	3/3
		DAB	2.46	0/3	0/3	0/3	3/3
523A1	BL	DBT	<0.01	3/3	3/3	3/3	3/3
		DAB	1.75	0/3	0/3	0/3	3/3
536	LL	DAB	18.2	0/3	0/3	0/3	3/3
537	LL	DAB	6.3	3/3	0/3	3/3	3/3
538	LL	DAB	1.23	1/3	1/3	0/3	3/3
539	BL	DAB	0.516	3/3	0/3	3/3	3/3
540	BL	DAB	3.28	0/3	0/3	0/3	3/3
542	BT	DAB	0.952	0/3	1/3	0/3	3/3
543	BT or BB	DAB	2.98	0/3	0/3	0/3	3/3

### (b) Genome-wide analyses

Of the 15 samples chosen for whole-genome sequencing, nine have sufficient coverage for whole-genome analysis (table 2). Of these samples, the depth of coverage ranges from 4x to 63×, with the per cent of the reference genome being covered at 5× ranging from 43% to 98%. The mapping statistics of the comparative data and a comparison of the mapping statistics for the four samples that underwent extraction using both methods are available in the electronic supplementary material, tables S2 and S3, respectively.

**Table 2.** Whole-genome analysis summary of samples sequenced in this study. (Note that reads generated from separate extractions were concatenated for samples 511, 515, 519 and 523A1.)

sample	origin	leprosy form	non-duplicate, uniquely mapped reads	average depth of coverage (X)	% reference covered > = 1×	% reference covered > = 5×	average fragment length	endogenous content^a^ %	branch	number of SNPs	non-synonymous coding SNPs
511	Samoa	LL	576 690	9.8	96	77	56	8.7	0	141	38
515	Hawaii	LL	635 824	10.1	95	73	52	15	5	101	20
516	Samoa	LL	635 186	13.1	98	94	67	64.2	0	172	54
517	Guam	LL	1 574 103	31.4	98	97	65	93.2	5	154	32
518	Hawaii	BL	2 899 280	63.2	98	98	71	80.5	0	184	57
519	Hawaii	BL	545 816	10.3	97	87	62	42.8	0	152	42
520	Hawaii	BL	1 616 046	32.7	98	97	66	94	0	183	56
523A1	Hawaii	BL	242 303	4.3	94	43	58	15.2	5	31^b^	7^b^
536	Guam	LLp	374 295	6.1	91	52	53	20	5	69^b^	12^b^
537	Northern Mariana Islands	LL	6308	0.1	8	0	44	0.4	–	–	–
538	Samoa	LL	1365	0	2	0	38	11	–	–	–
539	Northern Mariana Islands	BL	20 388	0.3	30	0	57	6.2	–	–	–
540	Northern Mariana Islands	BL	4691	0.1	7	0	54	1.3	–	–	–
542	Palau	BT	1589	0	2	0	53	5.7	–	–	–
543	Palau	BT or BB	1889	0	3	0	49	1.3	–	–	–

^a^Number of reads after mapping before duplicate removal/number of trimmed and merged reads.

^b^The low number of SNPs in samples 523A1 and 536 reflects the overall low coverage of those genomes at ≥ 5× depth of coverage.

The average fragment lengths of merged reads mapped to the *M. leprae* reference range from 38 to 71 bp for all 15 samples, and from 52 to 71 bp for the nine samples with an average depth of coverage greater than 4×. For the eight samples that were sequenced without UDG treatment, fragment misincorporation ranged from 1.5% to 3.5% at the terminal 3′ base and from 1.8% to 3.7% at the terminal 5′ base (electronic supplementary material, table S4). A visual comparison of a sample that was sequenced with and without UDG treatment can be seen in the electronic supplementary material, figures S1 and S2.

Using SnpEff, a range of 31 to 184 SNPs was identified in the new genomes presented here (table 2). Additionally, samples have between 7 and 57 non-synonymous SNPs in coding regions. A summary of SNPs and their effects can be found in the electronic supplementary material, table S5. Only five samples have unique SNPs located within genes, 515 (*n* = 1), 516 (*n* = 2), 517 (*n* = 3), 518 (*n* = 2) and 520 (*n* = 1); details of these unique SNPs can be found in the electronic supplementary material, table S6.

The genomes generated in this study were found to have all previously identified branch-defining SNPs for either branch 0 or 5 (electronic supplementary material, table S7) [3]. Additionally, the Pacific Island clade of four genomes within branch 5 (515, 523A1, 536, US57) was found to share four non-synonymous SNPs, and the Pacific Island clade of six genomes within branch 0 (511, S9-96008, 516, 518, 519, 520) was found to share 20 non-synonymous SNPs, including one in a gene associated with drug resistance (rpoB).

### (c) Phylogenetic analyses

**Figure 1.**
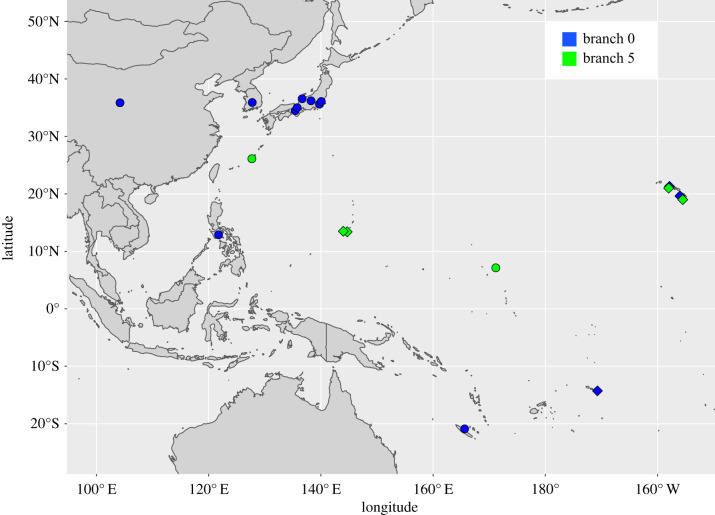
Map of the Pacific showing source locations of novel and previously sequenced *M. leprae* strains from branch 0 and branch 5. Novel strains from this study all fall within these branches and are shown as diamonds. Comparative data are shown as circles. (Online version in colour.)

**Figure 2.**
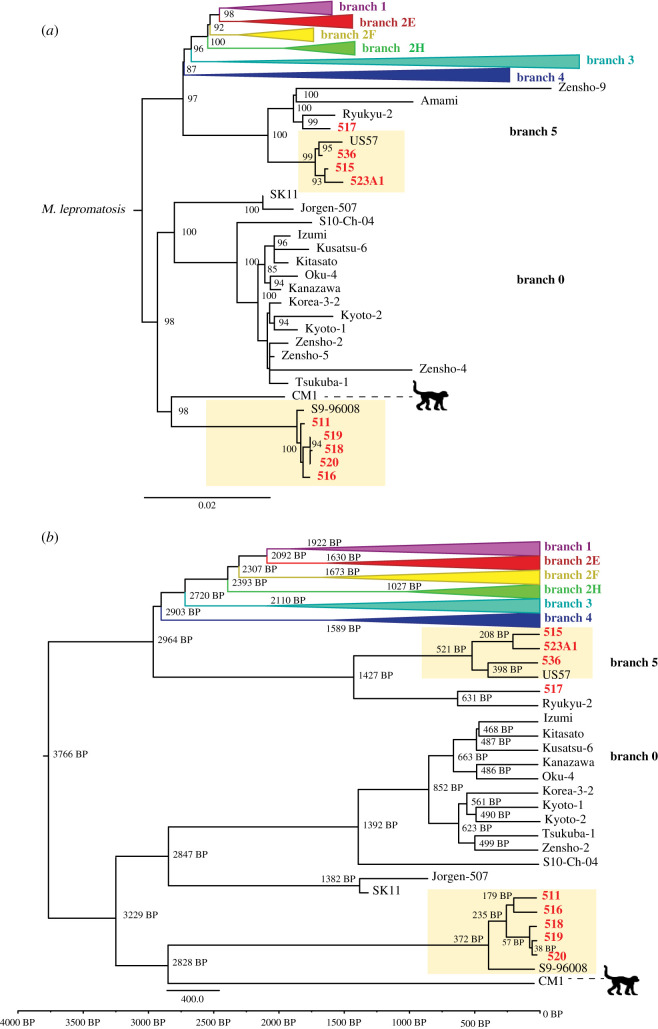
(*a*) Maximum-likelihood phylogenetic tree created using 2736 informative SNPs from an alignment of 164 comparative samples and the nine new genomes presented here; branch lengths are proportional to the number of substitutions, and the bootstrap values above 85 are present at nodes. Novel strains from this study are shown bold and in red. Pacific Island-specific clades are designated with beige boxes. (*b*) Bayesian phylogenetic tree based on 2184 informative SNPs as well as shared invariant sites calculated with BEAST 2.4.5 [59]. Median divergence times before present (BP) shown on main branch nodes. Novel strains from this study are shown bold and in red. Pacific Island-specific clades are designated with beige boxes. (Online version in colour.)

Phylogenetic analyses show that the new *M. leprae* genomes fall within the most basal branches, branches 0 and 5. Specifically, the genomes from Samoa (*n* = 2) fall onto branch 0, the genomes from Guam (*n* = 2) fall onto branch 5, and genomes from Hawaii fall onto branch 0 (*n* = 3) and branch 5 (*n* = 2) (figure 1). All of our phylogenetic trees reveal a topology that is consistent with previously published studies [3,4,11], as well as a consistent branch placement for the nine new genomes we introduce here (figure 2; electronic supplementary material, figure S3).

The inclusion of the Pacific Island genomes creates geographically associated substructure within branch 0. The Pacific Island genomes, including S9-96008 from New Caledonia, form a clade, as do the East Asian lineages and the medieval European lineages. Additionally, the branch 0 Pacific Island genomes are more closely related to a strain isolated from a naturally infected crab-eating macaque from the Philippines [58] than they are to other branch 0 strains. In branch 5, the two genomes from Hawaii (515 and 523A1) and one genome from Guam (536) are closely related to a strain that was isolated from the Marshall Islands (US57), while one genome from Guam (517) is more closely related to a strain previously isolated from the Ryukyu islands of Japan (Ryukyu-2).

### (d) BEAST analyses

The tMRCA for *M. leprae* strains was estimated to be 3766 y BP (3011 y – 4572 y 95% highest posterior density (HPD)) under the Bayesian Skyline model with a relaxed lognormal clock and a GTR substitution model (figure 2*b*). The mutation rate for *M. leprae* was estimated to be 3.255 × 10^−9^ (2.706 × 10^−9^ − 3.821 × 10^−9^, 95% HPD) substitutions site^−1^ y^−1^, which is slightly slower than previous estimates [3,4,58]. The Bayesian Skyline analysis run under a strict clock model with a GTR substitution model produced a tMRCA of 3878 y BP (3284 y – 4510 y BP 95% HPD) with a substitution rate of 3.101 × 10^−9^ (2.654 × 10^−9^–3.554 × 10^−9^, 95% HPD) substitutions site^−1^ y^−1^. The Bayesian skyline analysis run under a relaxed clock model with an HKY substitution model produced a tMRCA of 3765 y BP (2973–4623 BP 95% HPD) with a substitution rate of 3.221 × 10^−9^ (2.643 × 10^−9^–3.834 × 10^−9^, 95% HPD) substitutions site^−1^ y^−1^. Further analyses run using two demographic models using alignments without novel samples, as well as without low coverage 523A1 and 536 samples, had similar results (electronic supplementary material, table S8).

## 5. Discussion

Here, we report nine *M. leprae* genomes from the Pacific Islands. Phylogenetic analyses show that they all belong to branches 0 and 5, which previously have been identified in modern contexts only in the Western Pacific, including Japan, China, and the Philippines, and, interestingly, in medieval Hungary and Denmark [3,4,58]. Prior to this study, only two whole genomes from the Pacific Islands had been published, with sample US57 (Marshall Islands) falling within branch 5 and sample S9-96008 (New Caledonia) falling within branch 0 [3,4]. In addition to creating geographically associated substructure in branch 0, the Pacific Island genomes we sequenced tripled the number of whole-genome samples within branch 5.

Our tMRCA estimate of 3766 y BP (3011 y – 4572 y 95% HPD) is very close to that of Benjak *et al*. [4] and about 300–750 years younger than the Schuenemann *et al*. [3] estimate. Analyses completed with different demographic and substitution models also have estimates of tMRCA within decades of our initial estimate. The estimated rates of substitution, however, differ slightly, both with different demographic and substitution models and between other studies. The estimated substitution rate site^−1^ y^−1^ of 3.255 × 10^−9^ (2.706 × 10^−9^–3.821 × 10^−9^, 95% HPD) is similar but slightly slower than those estimated previously [3,4,11,67], with non-overlapping 95% HPD ranges in some cases [3,4,67]. Our analyses suggest a marginally younger tMRCA and slower substitution rate that is within the same magnitude as previous estimates. Despite these differences with previously published studies, our overlapping HPD ranges obtained when using multiple models and different combinations of samples indicate that our data are robust and informative (electronic supplementary material, table S8).

Our data suggest that *M. leprae* may have been in the Pacific Islands since the initial peopling of Remote Oceania and was possibly re-introduced during newly recognized subsequent migrations [68,69]. Remote Oceania was occupied beginning around 3000 BP by Austronesian-speaking people who originally expanded out of East Asia [68,70–72]. Ancient DNA data suggest that another migration wave from New Guinea nearly replaced these original inhabitants by 2300 BP [68,69]. Because the Pacific Island *M. leprae* genomes form a clade within branch 0, and the date of the last common ancestor of branch 0 clades is 3229 BP, it is possible that the Austronesian migration brought this basal lineage to Remote Oceania. This scenario also aligns with a South Asian origin of leprosy, which has been suggested elsewhere [6]. Because branch 5 strains share a common ancestor with branches 1–4 at 2964 BP and only consist of genomes from Pacific Islands, it is possible that these younger lineages were introduced during subsequent migrations into Remote Oceania.

The Pacific Island clade within branch 0 is differentiated from other branch 0 strains by a high number of non-synonymous SNPs, in contrast to the Pacific Island clade within branch 5 and the overall low genetic diversity of *M. leprae*, which probably reflects the older age of this clade. The low diversity within the branch 0 Pacific Island clade could reflect an introduction and radiation of these strains within the Pacific Islands around 350–400 years ago. The low divergence among these lineages, however, could be the result of sampling bias or genetic drift during infection and transmission across the Polynesian Islands during this time. Multiple lines of evidence, such as oral histories [73], geochemical analysis of traded stone tools [74,75], and population genetics of the Polynesian rat [76], demonstrate that voyaging among islands was common after initial settlement until approximately 1600 CE [74,77]. This cessation of inter-island voyaging roughly aligns with the age of the branch 0 Pacific Island clade and may have served as a bottleneck for *M. leprae* diversity. Nonetheless, the tight geographical clustering of clades, particularly within branch 0, coupled with the old age of the last common ancestor of Japanese and Pacific Island strains, strongly supports a more ancient introduction.

Despite palaeopathological evidence for leprosy in the Pacific Islands prior to 1000 CE, some researchers have suggested that leprosy was absent in the Pacific Islands until the nineteenth century CE and was introduced as a result of European and Japanese imperialism or large-scale Chinese migration throughout the region [38,41,78–80]. However, if strains were introduced during the Japanese imperial occupations beginning in 1890, we would not expect to see such deep divergence between Japanese and Pacific Island strains. Furthermore, we would expect the Pacific Island genomes to be more closely related to the genome from China (S10-Ch-04), instead of having diverged over 3000 years ago. Likewise, if European colonists introduced *M. leprae* to the Pacific Islands, we would expect at least some modern infections to be caused by branch 3 lineages, since (i) this branch was suspected to have been introduced to the Americas by European colonists, (ii) strains falling within branch 3 have been isolated from modern squirrels in the UK, and (iii) members of the branch have been identified in high frequency, along with branch 2, in medieval European skeletons [3,4,67]. Additionally, if strains were introduced in the sixteenth century during European exploration, then we would expect the Pacific Island strains in branch 0 to be more closely related to the ancient European strains within branch 0 (SK11 and Jorgen 507).

Although the timeline we propose based on the phylogeographical and evolutionary dating analysis is not corroborated by the archaeological record, this is probably owing to the poor preservation on tropical islands, as well as the paucity of large documented skeletal assemblages from these earlier time periods across Asia. Additionally, it is possible that these earlier lineages of *M. leprae* did not elicit the same skeletal response as they do today, obscuring the identification of the disease in the archaeological record. We also have a poor understanding of what animal reservoirs exist in this region and the extent of exchange among species. Though the Pacific Island branch 0 strains are closely related to one isolated from a crab-eating macaque from the Philippines [58], it is unclear when and in which direction the exchange occurred. Further sampling is clearly needed to expand our understanding of genetic diversity; however, in regions without sufficient archaeological evidence of leprosy and few surveys of potential animal reservoirs, clinical specimens, such as FFPE samples, are valuable resources for characterizing modern diversity of *M. leprae* in humans.

For this study FFPE samples allowed us to expand upon our knowledge of modern standing diversity in branches 5 and 0. Such samples have been successfully used in past *M. leprae* research [4,81], although both studies used a DNA extraction kit more specific to FFPE DNA. We found that DNA extraction protocols commonly used for ancient DNA were also effective. Our small comparison between the DAB and DBT extraction techniques found marginal improvements in coverage and mapping quality in the ancient DNA-specific DAB methods, but the limited sample size prevents a conclusive comparison. Interestingly, the DAB extracts had an increased extraction quantification of 2–50 fold over the DBT extracts but had fewer replicates amplifying for the qPCR assays. This could reflect the inclusion of more inhibitors through the DAB extraction or, because all *85B* assays on the DAB extracts were performed during the same experiment, an inefficient qPCR run. The small sample size of this study precludes definitive conclusions about which extraction method is best for FFPE material. The added heat treatment step and extended proteinase K treatment targeted DNA crosslinks and protein-DNA crosslinks, which are commonly induced during the formalin fixation step [82,83]. DAB methods and downstream bioinformatics pipelines can also address extensive DNA fragmentation found in FFPE samples, as well as the duplicates accumulated during the multiple rounds of amplification required for targeting low-concentration endogenous DNA [42,45]. Despite the difficulties of dealing with FFPE samples, methods are available to address their shortcomings and add a useful resource to answer further questions regarding *M. leprae* evolutionary history.

## 6. Conclusion

The phylogeographical and evolutionary dating analyses of nine new *M. leprae* genomes from the Pacific Islands suggest that *M. leprae* may have been introduced to Remote Oceania during the first human migrations at approximately 3000 BP, with re-introduction during subsequent migrations. Although a better understanding of *M. leprae* genomic diversity in Near Oceania and China is needed to support this hypothesis further, the data presented here strongly suggest a premodern introduction of leprosy into the Pacific Islands and refute its initial introduction either during European exploration or nineteenth century imperialism and colonialism.

## Supplementary Material

SI Figures 1 and 2

## Supplementary Material

SI Figure 3

## Supplementary Material

SI Figure 4

## Supplementary Material

SI Figure 5

## Supplementary Material

SI BST Analysis

## Supplementary Material

SI Table 1

## Supplementary Material

SI Table 2

## Supplementary Material

SI Table 3

## Supplementary Material

SI Table 4

## Supplementary Material

SI Table 5

## Supplementary Material

SI Table 6

## Supplementary Material

SI Table 7

## Supplementary Material

SI Table 8

## Data accessibility

All raw sequencing data generated for this project, including samples, extraction blanks and library preparation blanks, can be found under the NCBI BioProject accession PRJNA603842. For convenience, NCBI SRA accession numbers by sample can be found in the electronic supplementary material, table S1. Scripts are available at https://github.com/ACStoneLab/Mleprae.
