# Supplementary material for: Evolutionary history of Mycobacterium leprae in the Pacific Islands
Source: Philos Trans R Soc Lond B Biol Sci. 2020 Oct 5;375(1812):20190582. doi: 10.1098/rstb.2019.0582 (PMC7702798; doi:10.1098/rstb.2019.0582)
Supplement: SI Figures 1 and 2 [file rstb20190582supp1.pdf]

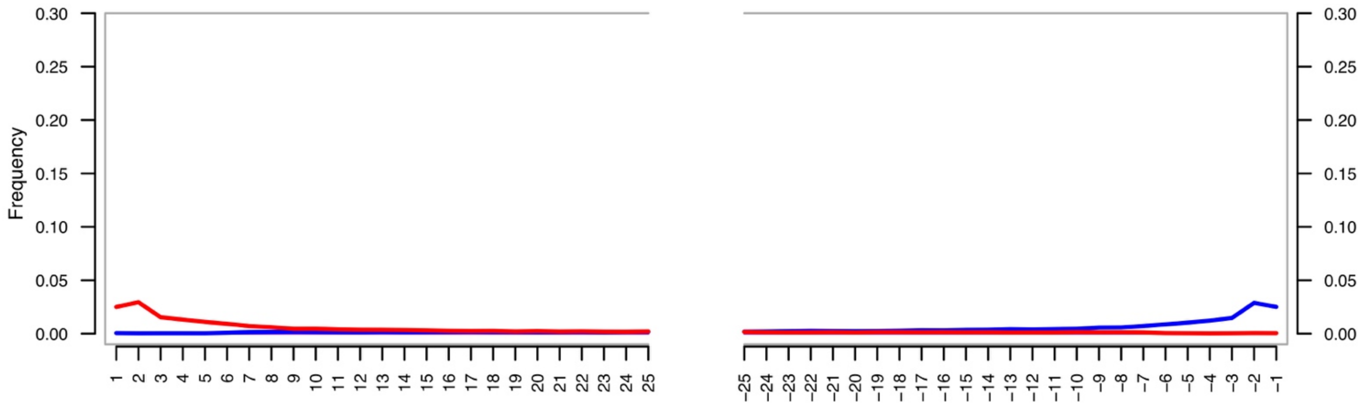

SI Figure 1. Sample 511 DBT extraction without UDG treatment fragment misincorporation plot showing the frequency of A -> G (left) and C -> T (right) transitions

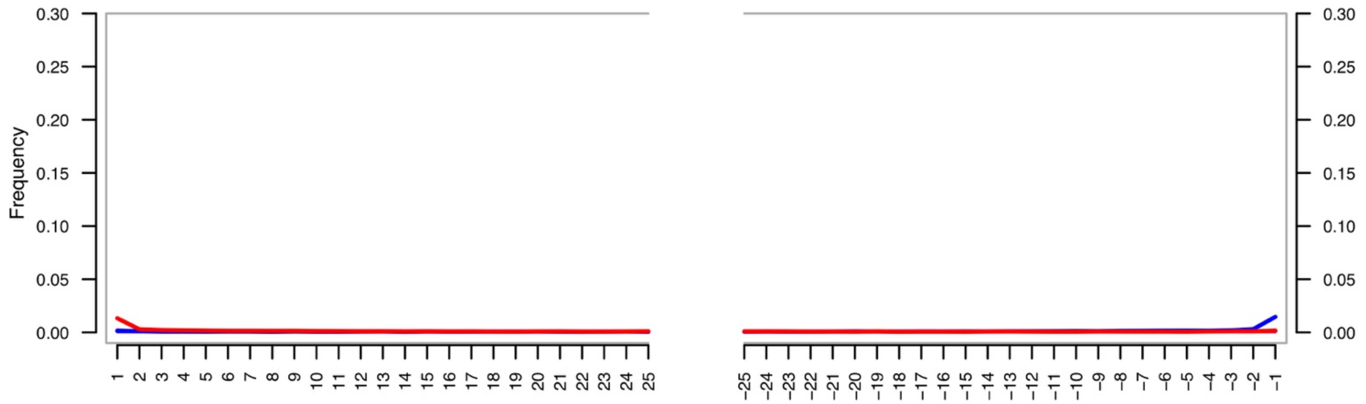

SI Figure 2. Sample 511 DAB extraction with UDG treatment fragment misincorporation plot showing the frequency of A -> G (left) and C -> T (right) transitions
