# Supplementary material for: Evolutionary history of Mycobacterium leprae in the Pacific Islands
Source: Philos Trans R Soc Lond B Biol Sci. 2020 Oct 5;375(1812):20190582. doi: 10.1098/rstb.2019.0582 (PMC7702798; doi:10.1098/rstb.2019.0582)

Paper Title: Evolutionary History of Mycobacterium leprae in the Pacific Islands

SI Figure 3: A maximum parsimony (MP) tree made using the subtree-pruning-regrafting inference model and a bootsrap test of phylogeny with 1000 replicates in MEGA7; Pacific Island clade highlighted in yellow.

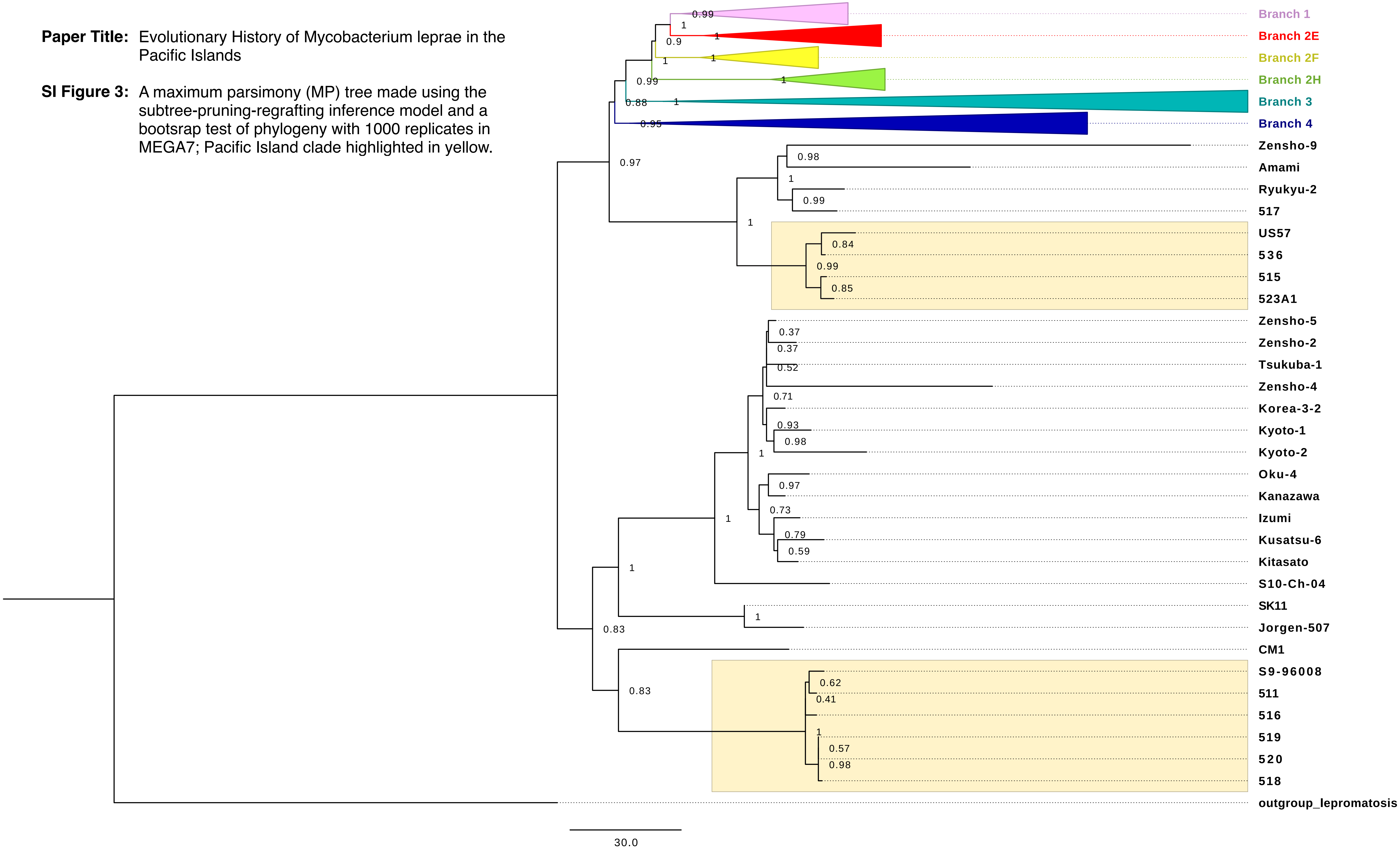

Supplement: SI Figure 3 [file rstb20190582supp2.pdf]
