# Supplementary material for: Evolutionary history of Mycobacterium leprae in the Pacific Islands
Source: Philos Trans R Soc Lond B Biol Sci. 2020 Oct 5;375(1812):20190582. doi: 10.1098/rstb.2019.0582 (PMC7702798; doi:10.1098/rstb.2019.0582)

Paper title: Evolutionary history of Mycobacterium leprae in the Pacific Islands

SI Figure 5. Bayesian skyline tree of novel samples and comparative data made using 2184 run under a relaxed lognormal clock and a GTR model.

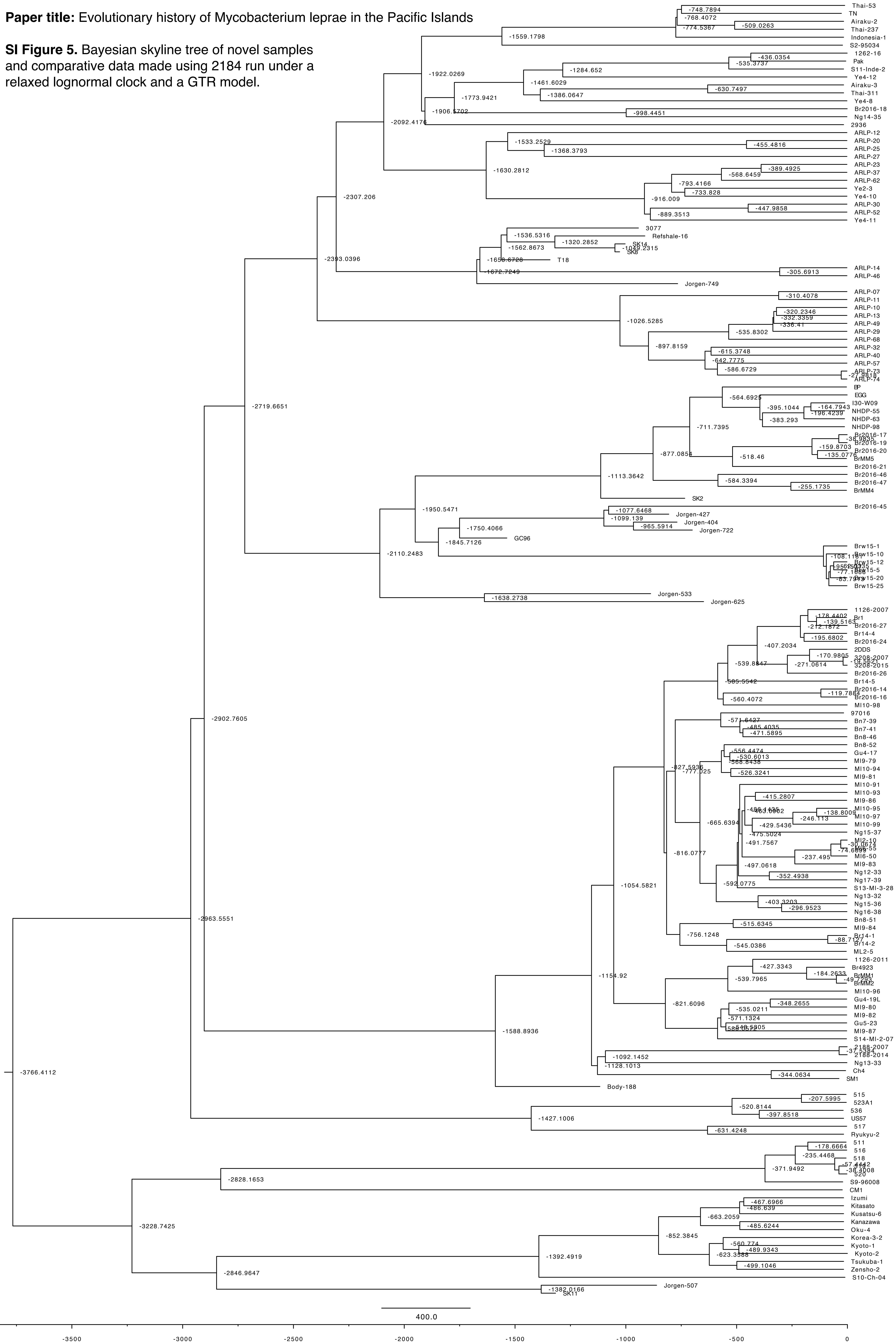

Supplement: SI Figure 5 [file rstb20190582supp4.pdf]
